# Adaptation Shifts Preferred Orientation of Tuning Curve in the Mouse Visual Cortex

**DOI:** 10.1371/journal.pone.0064294

**Published:** 2013-05-23

**Authors:** Jeyadarshan Jeyabalaratnam, Vishal Bharmauria, Lyes Bachatene, Sarah Cattan, Annie Angers, Stéphane Molotchnikoff

**Affiliations:** Université de Montréal, Département des Sciences Biologiques, Montréal, Canada; Tokai University, Japan

## Abstract

In frontalized mammals it has been demonstrated that adaptation produces shift of the peak of the orientation tuning curve of neuron following frequent or lengthier presentation of a non-preferred stimulus. Depending on the duration of adaptation the shift is attractive (toward the adapter) or repulsive (away from the adapter). Mouse exhibits a salt-and-pepper cortical organization of orientation maps, hence this species may respond differently to adaptation. To examine this question, we determined the effect of twelve minutes of adaptation to one particular orientation on neuronal orientation tuning curves in V1 of anesthetized mice. Multi-unit activity of neurons in V1 was recorded in a conventional fashion. Cells were stimulated with sine-wave drifting gratings whose orientation tilted in steps. Results revealed that similarly to cats and monkeys, majority of cells shifted their optimal orientation in the direction of the adapter while a small proportion exhibited a repulsive shift. Moreover, initially untuned cells showing poor tuning curves reacted to adaptation by displaying sharp orientation selectivity. It seems that modification of the cellular property following adaptation is a general phenomenon observed in all mammals in spite of the different organization pattern of the visual cortex. This study is of pertinence to comprehend the mechanistic pathways of brain plasticity.

## Introduction

In general, neurons in the sensory cortices respond preferentially to stimulus features eliciting maximal firing rates. Brain has a remarkable tendency to change its neuronal properties in response to the stimulus [1]–[5], though most of these original properties and connections are wired during the developmental critical period [6], [7].

It has been shown that the cortical neurons can adapt when a non-preferred stimulus is imposed on them for shorter or lengthier durations [8]–[18]. Adaptation leads to either attractive or repulsive shifts in neuronal tuning (orientation, spatial frequency, speed, motion direction). An attractive shift is a shift when the tuning curve of a neuron shifts toward the adapter, whereas a repulsive shift relates to the shift of the tuning curve away from the adapting stimulus. Moreover, investigations in cat and monkey visual cortices have shown that shorter adaptation durations lead to frequent repulsive shifts [10], [11], whereas longer and repetitive adaptations induce majority of attractive shifts [14]–[17].

Neurons in the visual cortices of higher vertebrates such as cats, monkey and humans are arranged in a columnar fashion [19], whereas cells are arranged in a salt-and-pepper fashion in mice and rats [20], [21]. Recently it has been shown that majority of neighboring cells in cat exhibit similar shifts (toward the adapter in majority) of tuning curves, and only 25% of groups of cells showed different direction of shifts following adaptation [13]. The salt-and-pepper organization implies that neurons with different orientation preferences are close to each other. The close proximity of these neurons may favor functional relations between them which consequently may lead to specifically different reactions following adaptation in comparison to cats and monkeys.

In this paper we report the effect of an imposed oriented sine-wave drifting grating on visual neurons in mice for a long time (12 min). Data show that attractive shifts are more frequent amongst orientation selective neurons. Interestingly, a number of cells that were originally poorly tuned (untuned) to orientation displayed selectivity following adaptation. We found that the shifts are not as systematic as in cats and monkeys, and this could be attributed to the salt-and-pepper functional organization of the cortex. Hence, it seems reasonable to postulate that columnar arrangement of neurons displays more methodical shift-pattern. Thus, we conclude that long adaptation leads to change in neuronal properties in response to the adapter, and this further consolidates the previous findings [1], [14], [15], [16] that forceful application of a stimulus modifies the neuronal selectivity.

## Materials and Methods

### Ethical approval

The animal preparation and recording procedures followed the guidelines of the Canadian Council on Animal Care and were approved by the Institutional Animal Care and Use Committee of University of Montreal. Animals were provided by the Division of Animal Resources of University of Montreal.

### Animal preparation

CD-1 strain adult mice aged from 9 to 11 weeks were used in this study. For recording from visual cortex the animals were anesthetized with 10% urethane (1.5 g/kg) injected intraperitoneally. Atropine sulphate (0.5 mg/kg) was administered subcutaneously to prevent accumulation of secretion in the trachea. Lidocaine hydrochloride 2% (Xylocaine) was applied at surgical and pressure sites as a local anesthetic. Pinch reflexes were used to assess the depth of anesthesia. The mice were then placed in a custom made stereotaxic apparatus allowing visual stimulation of the entire contralateral visual field and the skull was secured in a head holder, which eliminated head movement. The section of the skull and dura mater (2.5×2.5 mm) over the visual cortex was dissected out. Mystacial macro vibrissae were cut to preclude obstruction of the visual field. Corneal desiccation of the stimulated eye was prevented by applying a thin layer of silicone oil while allowing a clear optical transmission. Eye movements were negligible within the period of recordings. The unstimulated eye was closed.

### Electrophysiological recordings and stimuli

Evoked extracellular neuronal activity was recorded with a glass microelectrode filled with 0.9% NaCl inserted in V1. Both multi-unit and single-unit activities were recorded from superficial layers (<1 mm). The signal from the microelectrode was filtered (300 Hz–3 kHz), amplified, displayed on oscilloscope and audio-monitored. Then multi-unit activity was digitized and recorded with data acquisition software (Spike 2, Cambridge Electronic Design, CED Limited, Cambridge, England). The off-line spike sorting method was based on cluster classification in reduced space. The stability of each cell's activity across conditions was verified qualitatively by visual control of the clusters disposition, the waveforms shape and auto-correlograms showing a total depression at its center. As we began by recorded multi-unit electrical activities, no attempt was made to stimulate the receptive fields. In mice the excitatory receptive field is rarely smaller than 10° [23]. The stimulating grating covered ±30° horizontally and ±30° vertically of the mouse monocular field.

Stimulation was monocular. For the entire study, visual stimuli were drifting full-contrast square wave gratings generated with a VRG Volante 34020 graphic board (Vision Research Graphics, New Hampshire, USA). Stimuli were presented on a 21-in. monitor (60 Hz refresh rate, Mitsubishi FHS6115SLK Color Display Monitor, Tokyo, Japan) with 1024×512 pixels placed 28.5 cm from the mouse's eye. Temporal frequency and velocity were constant at 0.07 cycles/deg and 4 deg/s respectively. These parameters were maintained constant throughout the duration of the experiment. The center of the monitor was positioned at about 45° azimuth 0° elevation.

### Visual stimulation protocol

Six orientations equally spaced were selected and used for this study covering a span of 90°. Test orientations were presented in a random order. Each oriented stimulus was presented in blocks of 25 trials lasting 4.1 s each with a random inter-trial interval (1.0–3.0 s) during which no stimulus was presented. We recorded peri-stimulus time histograms (PSTH) of multi-unit activity.

Once control orientation tuning curves were characterized, an adapting stimulus was presented continuously for 12 min. The adapting stimulus was a quasi full field drifting grating whose orientation was 45°. During this adaptation period no recordings were performed. Immediately after adaptation, orientation tuning curves were measured starting with the adapting orientation while the remaining orientations were recorded in a random order. Following a recovery period of 60 min, another orientation tuning curve measurement was performed.

### Data analysis

Single cells were sorted out off-line from the multi-unit activity recorded during data acquisition. Evoked responses were derived from peri-stimulus time histograms which provided cellular firing rates. Orientation tuning curves were constructed from raw data and fitted using the Gaussian function. This allowed us to determine the preferred orientation of neurons with precision and then measure shifts in orientation preference. Since orientation tuning is best described with Gaussian-like functions, we fitted our raw data with the von Mises function [22]. This allowed us to determine the preferred orientation of neurons and then measure shifts in orientation preference. The von Mises function is defined as: 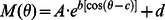 where A is the value of the function at the preferred orientation, c, and b is a width parameter. An additional parameter, d, represents the spontaneous firing rate of the cell [22], [24]. We analyzed cells whose tuning fitted well von Mises function over the (90°) range of sampled orientations. The above calculations are necessary because tuning curves derived from raw data may be imperfect in determining the preferred orientation as the interval between the presented orientations is relatively large. A fit was considered satisfactory if it accounted for at least 80% of the variance in the data.

It was also necessary to ensure that cells in our sample were properly tuned for orientation. We measured an orientation selectivity index (OSI) by dividing the firing rate at orthogonal orientations (baseline of the tuning curves) by the firing rate for the preferred orientation, and subtracting the result from 1 [25]. The closer the OSI is to 1, the stronger the orientation selectivity because it means that the ratio baseline/preferred orientation is small, implying that the firing rate at the preferred orientation is much greater than the baseline-firing rate. No significant difference was found between OSI values measured from raw or fitted data. The figures we present in the results section are from raw and fitted data.

## Results

We investigated the orientation tuning curves of V1 neurons following the adaptation protocol. Neurons were sorted from the multi-unit activity recorded in the primary visual cortex of anesthetized mouse. A total of 108 cells were sorted out and are shown in Table 1. In the first step we presented gratings randomly at varied orientations to generate orientation tuning curves. Following twelve minutes of adaptation at 45°, tuning curves were fitted again. A recovery from adaptation effects was carried out about sixty minutes after adaptation phase. Orientation tuning curves were fitted using a Gaussian function (see Material and methods).

**Table 1:** Classification of cells after adaptation.

	Attractive	Repulsive	No-shift	Other (67.6%)		
Total cells, n = 108 cells	14 (13%)	4 (3.7%)	17 (15.7%)	22 UT (20.4%)	47 UU (43.5%)	4 TU (3.7%)

Legend: UT: Untuned to Tuned, UU: Untuned to Untuned, TU: Tuned to Untuned.

Out of investigated 108 cells 14 (13%) cells were characterized with attractive shifts, whereas tuning curves of 4 (3.7%) cells repelled away from the adapter. 17(15.7%) neurons displayed no significant shift, that is, they retained their original preferred orientation. Other cells were categorised as follows: 22 UT neurons (20.4%), that is, untuned neurons that showed no preferred orientation tuning curves (OSI = 0.10) before adaptation, but exhibited tuning after an adaptation of 12 min (OSI≥0.70); 47 cells were UU neurons (43.5%), that is, neurons which displayed no preferred orientation prior and post-adaptation; and finally 4 TU neurons (3.7%) which were tuned before adaptation, but behaved with no orientation selectivity post-adaptation. In summary this table indicates that out of all originally orientation selective neurons which changed their tuning after adaptation, most of them responded with attractive shifts. It also demonstrates that adaptation may have varied effects on neurons. It may induce orientation selectivity in cells originally lacking an optimal orientation, and may even potentiate originally tuned neurons to lose their selectivity. Finally, a large proportion of cortical cells in V1 in mice exhibited poor tuning and was insensitive to adaptation.

### Adaptation-induced plasticity of orientation tuning curves

In all cases the adapting orientation was applied on the flank of the tuning curve, the offset generally was 30° from the optimal orientation. Curve fits were generated for each of the 25 trials and Student's t-test was done between control and adaption conditions to evaluate the significance of orientation shifts on the single cell basis.

**Figure 1:**
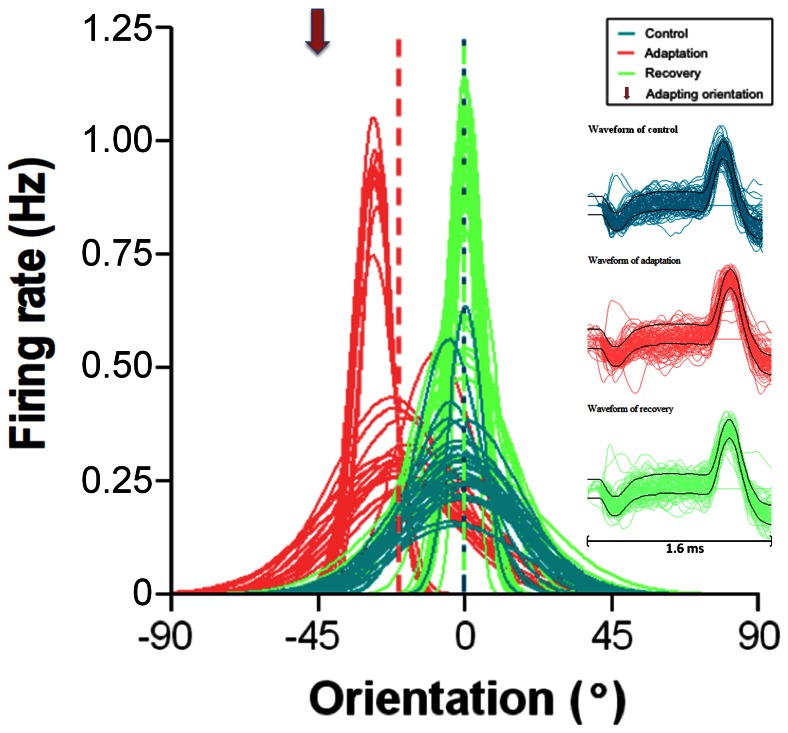
Typical example of an attractive shift. Twenty-five presentations of each orientation. Gaussian fits and orientation are normalized in this and other figures: optimal orientation is marked zero. Vertical broken lines indicate optimal orientations. Downward arrowhead indicates adapting orientation in this and all figures. Horizontal Right inserts show spike waveforms recorded during each phase of the recordings. Bar equals 1.6 ms.

Figure 1 describes a typical example of an attractive shift (toward the adapter). Blue tuning curves correspond to values before adaption. Since this study is centered on shifts of tuning curves and to ease comparison between cells, optimal orientations were normalized to 0°. These curves show that although response magnitude fluctuates from one presentation to the next (in the example range 0.14 Hz to 0.64 Hz) the jitter for optimal orientation is small (<5°). Initial optimal orientation was 90° (normalized to 0°). Immediately after twelve minutes of adaptation, the orientation selectivity was tested again with identical stimuli (red tuning curves). Following adaptation, the preferred orientation shifted in direction of the adapter (arrow head adapter oriented at −45°). The acquired new optimal orientation is −20°. Thus the cell showed a significant attractive shift of 20° (optimal orientation, paired sample two-tailed t-test, P<0.0001, interrupted vertical lines represent the averaged optimal orientation). In addition, this change in orientation selectivity is accompanied by an increase (26.7%) of the responsivity. About sixty minutes (recovery period) after the end of adaptation protocol, the original preferred orientation was reinstated (illustrated by the green tuning curves). On the right (Figure 1), waveforms of action potentials recorded during each step of this recording are displayed. Identical waveforms ascertain that responses were produced by the same unit during all phases of the study.

**Figure 2:**
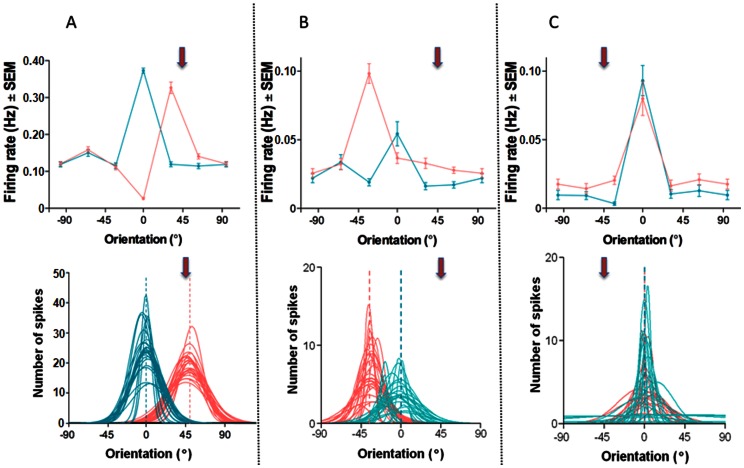
Three examples of orientation shifts. From left to right (**A**) attractive, (**B**) repulsive and (**C**) no shift. Upper row: Raw data, error bars indicate SEM. Bottom row: Gaussian fits, same cells as in the upper row.

Figure 2 illustrates three typical behaviors of neurons after adaptation. The upper row displays the tuning curves as derived from raw data (error bars indicate SEM). Lower plots illustrate trial-by-trial Gaussian fitted orientation tuning curves pre and post-adaptation. The first cell (Figure 2A) showed a significant attractive shift of 49° following adaptation (paired sample two-tailed t-test, P<0.0001). The second cell (Figure 2B) showed a significant repulsive shift of 35° after adaptation (paired sample two-tailed t-test, P  = 0.0032). The third cell (Figure 2C) is an example of a typical refractory neuron that shows no significant shift. Indeed the preferred orientation is unchanged in spite of variability of the evoked discharges from trial to trial [26], (paired sample two-tailed t-test, P>0.1).

Moreover, these plots underline the fact that there is a strong decline of responses evoked by the original preferred orientation, regardless of the direction of the shifts. In these examples the magnitude of responses to the initial preferred orientation declined to the level of the remote flank orientations. In addition these examples illustrate the response enhancements of novel peak responses. Additionally, activity driven by orientations outside the range of the adapter and original optimal orientation is unchanged (Figure 2), suggesting that imposing a particular orientation for several minutes impacts mechanisms influencing original and the novel optimal orientations. These data point toward the fact that adaptation effects are mostly constrained around the adapter and the original optimal orientations. It signifies that the peak shifts are not the consequence of the global modulation of neuronal activity.

The mean shift was calculated for a subset of 18 cells (originally tuned cells showing significant shifts). For this computation, cells displaying attractive and repulsive shifts were pooled together to assess the overall significance of shift-magnitude. Shifts in orientation preference of amplitude larger than 5° were all significant (P<0.05, t-test) and represented 89% of the cells (16/18 cells). The absolute average amplitude of significant shifts was 53.64°±8.40°. The mean amplitude of significant attractive shifts was 60.91°±12.61° and 20.25°±0.74° for repulsive shifts respectively.

**Figure 3:**
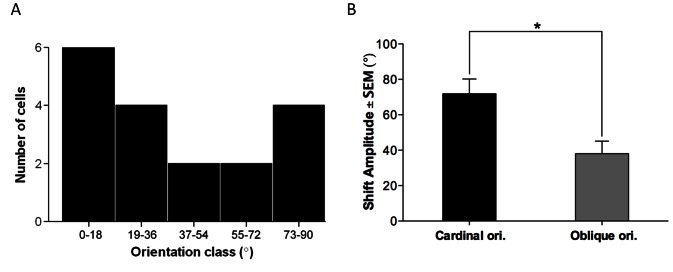
Distribution of orientation-class preference and shift magnitude significance. (**A**) Neurons divided into five orientation classes of 18°. Vertical and horizontal orientations dominate (that is cardinal orientations). (**B**) The relationship between shift-magnitude and significance level indicates that cardinal orientations have larger shift than cells whose orientation is oblique.

Figure 3A shows the distribution of orientation-class preference for this pool of neurons. The population of neurons was divided into five orientation classes of ±18°. Similarly to cats, vertical and horizontal orientations seem to dominate this distribution (that is cardinal orientations). The survey shown in Figure 3B indicates that cardinal orientations exhibit bigger shifts 72±8.3° than oblique orientations. The displacement of the peak of the tuning curve in this case averaged 38±7.0°, that is, almost half of the shift-magnitude induced at cardinal orientations (p = 0.0287, t-test). However, this difference may be attributed to frequent application of an oblique adapter (45°).

### Orientation tuning curve emergence

**Figure 4:**
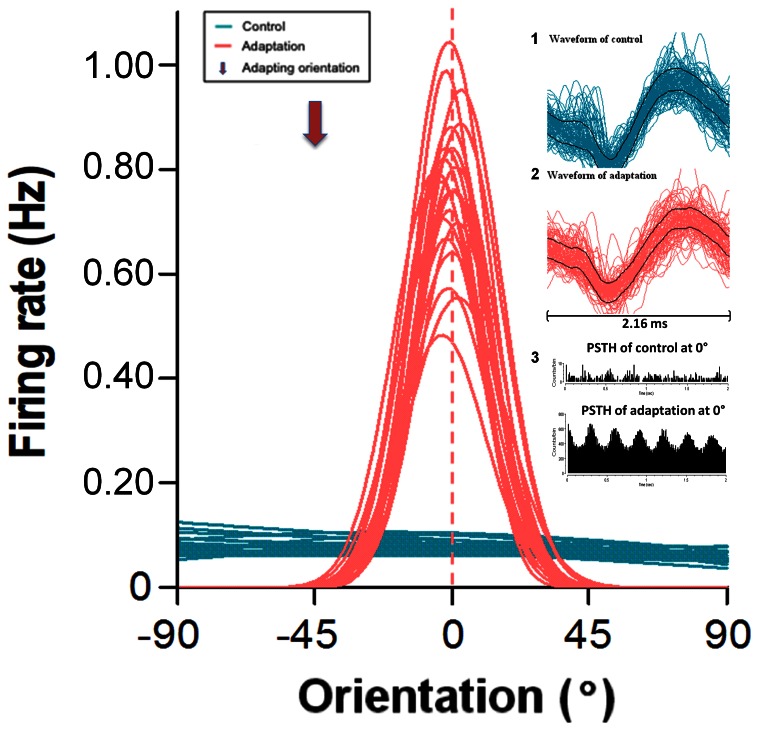
Example of a neuron untuned to orientation. Prior to adaptation (in blue) no orientation evokes a stronger response. Following adaptation (in red) a clear optimal orientation emerges. Right inserts, tracing 1 and 2 show spike waveforms recorded during each phase of the recordings, bar equals 2.16 ms. Tracings in 3 illustrates PSTHs of evoked responses prior to (upper) and following (lower) adaptation respectively.

Contrary to what is observed in cats and monkeys where most of the cells exhibit orientation selectivity (significant orientation tuning curve) in V1, in mice a fairly large (>50%) proportion of neurons failed to show selectivity for a specific orientation [23]. For instance, in Figure 4 the unit responded to any orientation with an almost equal firing rate (approximately 15 spikes per trial) to all presented orientations (blue). The weak responsiveness is shown in the upper PSTH (Figure 4, tracing 3) Quite interestingly, and unexpectedly, following a period of twelve minutes of adaptation to a grating oriented at 45°, the same cell displayed a robust orientation tuning curve whose preferred orientation was horizontal (0°) and the OSI  = 0.92. The PSTH in tracing 4 illustrates the firing rate corresponding to the acquired optimal orientation. It is important to underline that this particular unit was recorded simultaneously within the same cluster of neurons that yielded the cell in Figure 2A. Thus, although both units were close to each other, only one neuron disclosed a typical tuning curve prior to adaptation. After adaptation both cells showed orientation tuning curves which indicates that the second cell (Figure 4) acquired this property through adaptation.

### Effects of adaptation on responses magnitude

**Figure 5:**
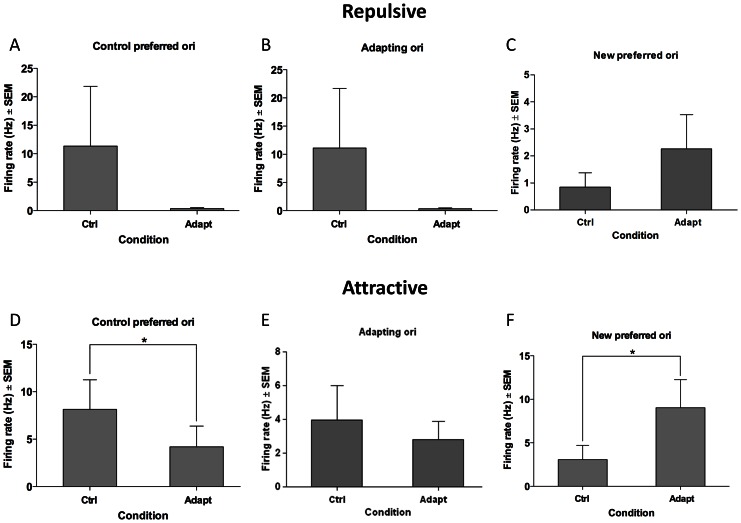
Comparative response modulations induced by adaptation. Upper row: repulsive shifts. Lower row: attractive shifts. Three responses are computed: Original preferred, responses to adapter, new preferred. Star indicates significant level p<0.05. Abbreviations, Ori: orientation, Ctrl: control, Adapt: adaptation

The average adaptation-induced response modulations are compared in Figure 5. The quantification is focused on three responses: the initial preferred response, the response to the adapter and finally the acquired optimal response. The upper row (Figure 5A–C) shows results related to repulsive shifts. These computations indicate that responses evoked by the original optimal orientation are considerably diminished (−96.83%). This decline of initial optimal firing is recorded quite systematically, and has been reported in previous studies carried out on other mammals [15], [24]. In addition, the evoked discharges in response to the adapting orientation also decreased by 96.94%. Thus, applying an orientation up to 90° off the preferred axis of orientation depresses responses on the flank of the tuning curve facing the adapter. By contrast, responses on the opposite flank relative to the adapter are augmented although this increase is insignificant, (62.72% % P>0.01). The global result however is that the repulsive shift is attributed to depression of responses on the slope of tuning curve facing the adapting orientation.

In comparison, the profile of response modulations for attractive shifts is quite different following adaptation (Figure 5D–F). Responses evoked by the original optimal orientation are strongly depressed (−48.47% p<0.05, t-test), in a fashion similar to repulsive shifts (see above). But in contrast to the repulsive shifts, responses produced by orientations close to the adapter are significantly enhanced leading to attractive shifts. Indeed, the acquired preferred orientation (Figure 5F) is the result of an average increase by 66% (p<0.05, t-test) in the firing rate evoked by orientation close to or corresponding to the adapter. This dual effect: decline of evoked responses to the initial optimal orientation and enhancement of discharges on the adapting flank may be the result of a push-pull process suggested for cats and monkeys [15], [24], [27], [28] that underwent similar experimental protocol.

## Discussion

Our results suggest that as for other mammals with eyes in frontal plane, mice cortical cells shift their peaks of orientation tuning curves in response to adaptation. This appears particularly interesting since these animals lack a columnar organization of the orientation domains as demonstrated in cats and monkeys. As a matter of fact investigations have suggested that mouse cortex exhibits salt-and-pepper type of organization for orientation, that is, a large spectrum of axes of orientations are interspersed over a small cortical distance; instead of a progressive tilt of orientation preference as recording electrode moves tangentially in shorter steps from one column to the next [7], [19].

Thus, in spite of this organizational difference orientation tuning curves are susceptible to relatively long period of adaptation: cells adopt new preferred orientations. Globally these results suggest that such shifts are a general property that would be applicable to all mammals. A general rule may be proposed for mammals: imposing a non-preferred stimulus for several minutes induces a modification of the preferred stimulus that was established since birth or during the critical period. Our results are quite synonymous with the data recorded by Frenkel et al., 2006 [12] describing selective response potentiation. This facilitation of responses relates to selective increase of responsiveness following corresponding exposure to the grating stimuli. In addition to orientation [29], other properties such as spatial frequency [9], [17], speed [24], direction [5], [30]–[33], patterned plaid [34], ocular dominance [35] are also triggered following frequent presentation of appropriate stimuli. Similar data have been reported in the auditory system [36], [37].

This type of plasticity may be explained with results for mice reported by Jia et al., 2010 [38]. Using double high-speed two-photon imaging and electrophysiological recordings, it has been demonstrated that a single dendritic branch receives synaptic connections from neurons selective to different orientations [38], [39]. Such large range indicates that many or all orientations may be efficient in exciting a single cortical cell. These studies demonstrate that each contact generates synaptic response as measured through calcium fluxes. Therefore, activating an alternate group of synapses shifts the optimal orientation. Because weak responses evoked by non-preferred orientation (flank orientation) are barely modified by adaptation, it seems that adaptation affects almost exclusively the initial optimal orientation (decline) and the new acquired orientation (facilitation). It has been suggested that this dual modulation is attributed to a push-pull mechanism [15], [24], [28], [40].

### Methodological considerations

It is unlikely that response modulations reported in the present paper are attributed to a random and sudden surge of electrical activity. Data show that flank orientations remained unchanged although these responses were weak. In addition evoked responses to the adapter are enhanced while responses to the initial preferred orientation are decreased. Such dual effects are irreconcilable with a uniform and global fluctuation of firing rates. Also, response modifications are roughly constrained around the adapter and the original optimal orientation. It is worth to signal that orientations are applied randomly. It may be argued that orientation tuning curves vary in relation to cell's discharge variability or stimuli dimensions. However, as shown in the present paper although response magnitude changes from trial to trial, the jitter of optimal orientation remains quite small. Since adapting orientation is applied uninterruptedly for twelve minutes, it may be argued that our results are due to change of luminosity induced by prolonged adaptation. This eventuality is unlikely. Shou et al., 1996 [41] reported that prolonged exposure to drifting gratings induces a global decline of responsiveness in most cells. In addition, facilitation of responses was a rare occurrence and no tuning shifts were recorded [42]. Finally, it is important to mention that firing increase makes it unlikely that neuronal fatigue is associated in adaptation mechanisms. In cats it has been shown that orientation change is not occurring at the LGN (lateral geniculate nucleus) level since cells at this level are unoriented. Thus, this sort of plasticity originates at cortical level [9], [10]. Finally, in the salamander retina [43] and cats adaptation to contrast modulates evoked responses. However, these changes are occurring in a few seconds, consequently they are not comparable to modulation observed in the present study.

### Comparison with other mammals

Several investigations report orientation attractive and repulsive shifts [1], [14], [15], [27]. Equally, in adult and mature cortices, other visual properties demonstrated changes following various adaptation protocols. Although the presented results are similar to those obtained in other species, several differences are noteworthy.

In mice a large proportion of cells are poorly tuned [23], and also in our study in which we employed a quasi full field presentation of gratings. We did not stimulate local receptive field as multi-unit recordings were carried out. However after adaptation the same cells exhibited a classical tuning curve even though the same stimuli were applied. Such emergence of orientation tuning curves was unreported in cat's study, may be because in cats most cortical cells are tuned to orientation in V1. This lack of orientation selectivity in mice may be attributed to the fact that cortical cells are connected by afferent axons displaying a large range of orientations [38]. Thus there is no bias favoring one orientation. Adaptation would induce a preference allowing one orientation to strengthen its drive over the remaining inputs. This argument seems reasonable for attractive shifts as the highest changes are close to the adapter. Alternatively, as we used large stimuli adaptation to one orientation, and this may cause a disinhibition of surround suppression which in turn results in an attractive shift. The same reasoning is conflicting with repulsive shifts. One may assume that the adapter weakens the inputs driven by the latter, which in turn facilitates responses on the opposite side of the tuning curve. Such description was advanced for repulsive shifts in cats [14], [15] and monkeys [24], [27].

It has been suggested that tuning function of individual cells is the result of converging and overlapping inputs. In mice this situation may be the result of an absence of clear and extensive columnar organization. Therefore cells with different preferred orientations are close to each other. This assumption is supported by above cited data reported in Jia et al., 2010 [38]. Such arrangement favors large shift of synaptic equilibrium depending on the stimuli conditions. Another difference with other animals deserves attention. Cells lacking orientation selectivity demonstrate a clear tuning curve after adaptation. Such emergence of orientation selectivity was unexpected. However, it is compatible with previous explanation stating that a single neuron receives a large spectrum of oriented inputs and following a forceful application of one particular orientation favors synaptic excitation associated with the adapter which leads to the emergence of an orientation tuning curve. Furthermore, this statement is in line with a recent report of Makino and Malinow [44] who identified grouped synaptic changes induced by experience at individual dendritic branches in the barrel cortex. They showed changes of synaptic spines in a grouped manner. In parallel, it has been demonstrated that exposing mice to grating stimuli of one orientation produces increase of responses to the applied stimulus [12], [45]. Hence our data suggest that synaptic modifications are occurring within relatively brief time window.

In conclusion, the results of the current paper are reasonable to suggest that despite the cortical organization, the neurons in either columnar or salt-and-pepper fashioned mammals exhibit behavioral shifts in response to longer adaptation periods. Nevertheless, the shift pattern in column-fashioned animals is more systematic than in mammals with salt-and-pepper orientation map.
